# Desmoid-type fibromatosis of the breast in a male patient following cosmetic surgery: a rare case report

**DOI:** 10.3389/fonc.2024.1438050

**Published:** 2024-12-17

**Authors:** Diana Carolina Correa Sandoval, Javier Gonzalez Reyes, Diego Alberto Guajardo Nieto, Jose Luis Guzman Murguia

**Affiliations:** ^1^ Vicerrectoria de Ciencias de la Salud, Universidad de Monterrey, San Pedro Garza Garcia, Nuevo Leon, Mexico; ^2^ Departamento de Enseñanza de Hospital Angeles Valle Oriente, San Pedro Garza Garcia, Nuevo Leon, Mexico; ^3^ Angeles Breast Center, Hospital Ángeles Valle Oriente, San Pedro Garza Garcia, Nuevo Leon, Mexico

**Keywords:** fibromatosis, male breast, malignancy, surgery, rare

## Abstract

**Background:**

Desmoid-type fibromatosis of the breast is a rare, benign, but locally aggressive tumor that typically affects women. Its presentation in male patients is exceedingly rare, and even more so following a cosmetic procedure such as liposuction. This case report describes a unique presentation of breast fibromatosis in a male patient, who developed the condition after undergoing liposuction for cosmetic purposes to define the pectoral area. The case highlights the diagnostic challenges it poses due to its malignancy-like appearance on imaging.

**Materials and methods:**

A 28-year-old male patient presented with a right palpable mass in the breast following a liposuction procedure aimed at enhancing pectoral definition. Imaging studies, including ultrasound and MRI, raised suspicion of a malignancy. However, histopathological analysis from a core needle biopsy revealed fibromatosis. The patient underwent surgical excision of the tumor, and histological evaluation confirmed the diagnosis of benign desmoid-type breast fibromatosis.

**Results:**

Histopathological examination revealed benign fibromatosis with no evidence of malignancy. The tumor was completely excised with clear margins, and the patient has had no signs of recurrence during the follow-up period.

**Conclusions:**

This case highlights the rare occurrence of breast fibromatosis in a male patient following liposuction for cosmetic purposes. Given its ability to mimic malignancy on imaging, early diagnosis and complete surgical excision are essential for effective management and to prevent recurrence.

## 1 Introduction

Desmoid-type breast fibromatosis is a rare, benign condition originating from fibroblastic or myofibroblastic cells, accounting for only 0.2% of all breast tumors, and is most commonly seen in females (1). This condition is often related to prior breast trauma or local surgical intervention, and despite its benign nature, it is known to proliferate aggressively (2). Imaging findings, particularly on ultrasound, typically show an irregular hypoechoic mass that closely mimics breast carcinoma (2).

We present a rare case of desmoid-type breast fibromatosis in a male patient who developed the condition following thoracic liposuction performed for aesthetic reasons to define the pectoral area. To date, only eight cases of male breast fibromatosis have been reported in the literature (1). The rarity of this presentation, combined with the potential for the lesion to simulate malignancy on imaging, highlights the importance of including desmoid-type breast fibromatosis in the differential diagnosis of atypical breast tumors that can mimic carcinoma.

## 2 Case report

A 28-year-old male patient with a surgical history of liposuction for aesthetic reasons at 23 years of age presented with a painless palpable mass in the right breast. This case is particularly rare due to the occurrence of desmoid-type breast fibromatosis in a male patient, a condition predominantly found in women, and its association with a prior cosmetic procedure. He had a family history of breast cancer, as his maternal aunt was diagnosed with breast cancer at the age of 32 and is currently alive. The patient had no history of radiation exposure or other significant medical conditions. He was not taking any regular medications and reported no tobacco or alcohol use. He maintained an active lifestyle and had no other risk factors for breast malignancy.

He presented to our clinic with a painless palpable mass in the right breast gland. Upon physical examination, no signs of edema, erythema, or hyperthermia were observed. A firm, mobile mass measuring 1 cm was palpated at 10 o’clock, 7 cm from the nipple, with no abnormalities detected in the axilla. The patient reported no associated pain, nipple discharge, or changes in skin texture. The left breast gland and axilla were normal. No systemic symptoms such as fever, weight loss, or fatigue were reported.

**Figure 1:**
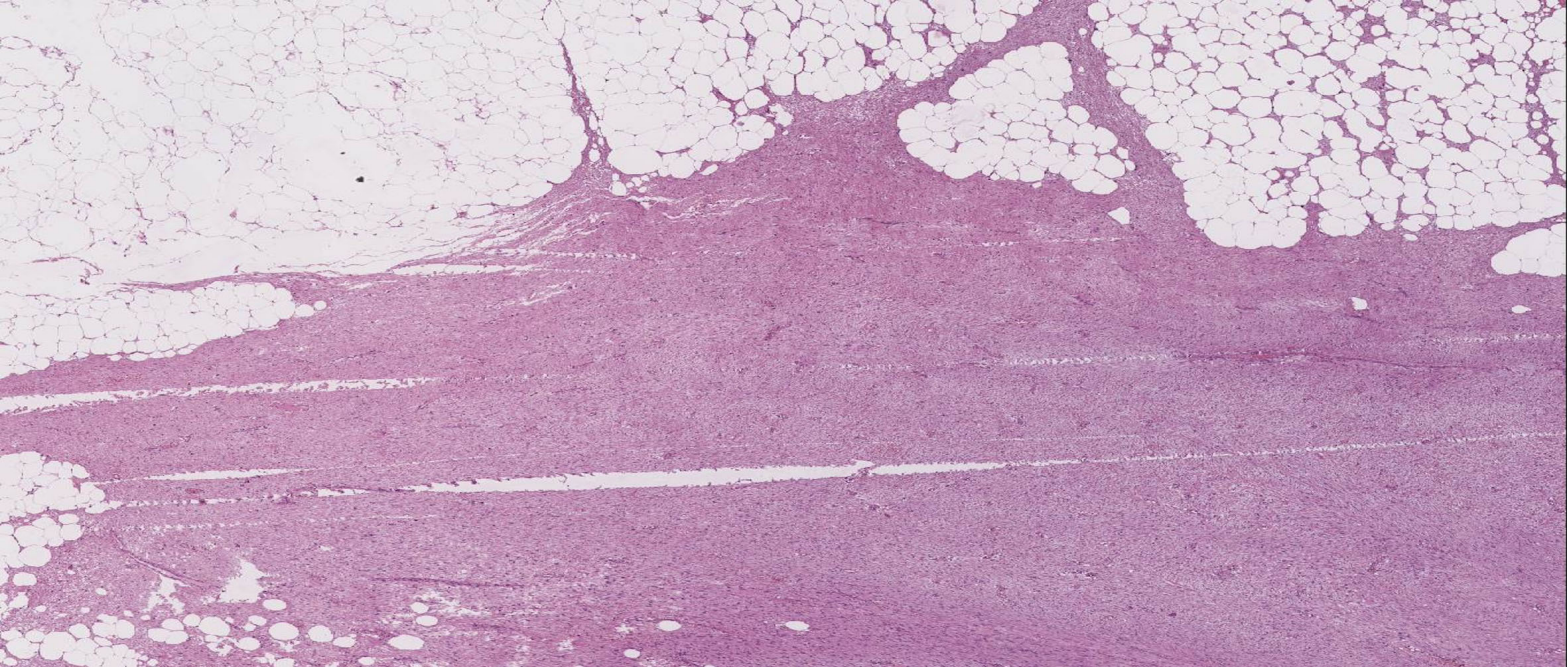
Image reveals a neoplastic, solid and encapsulated lesion of soft tissues. The lesion has ill-defined margins and infiltration (H&E, 20x).

**Figure 2:**
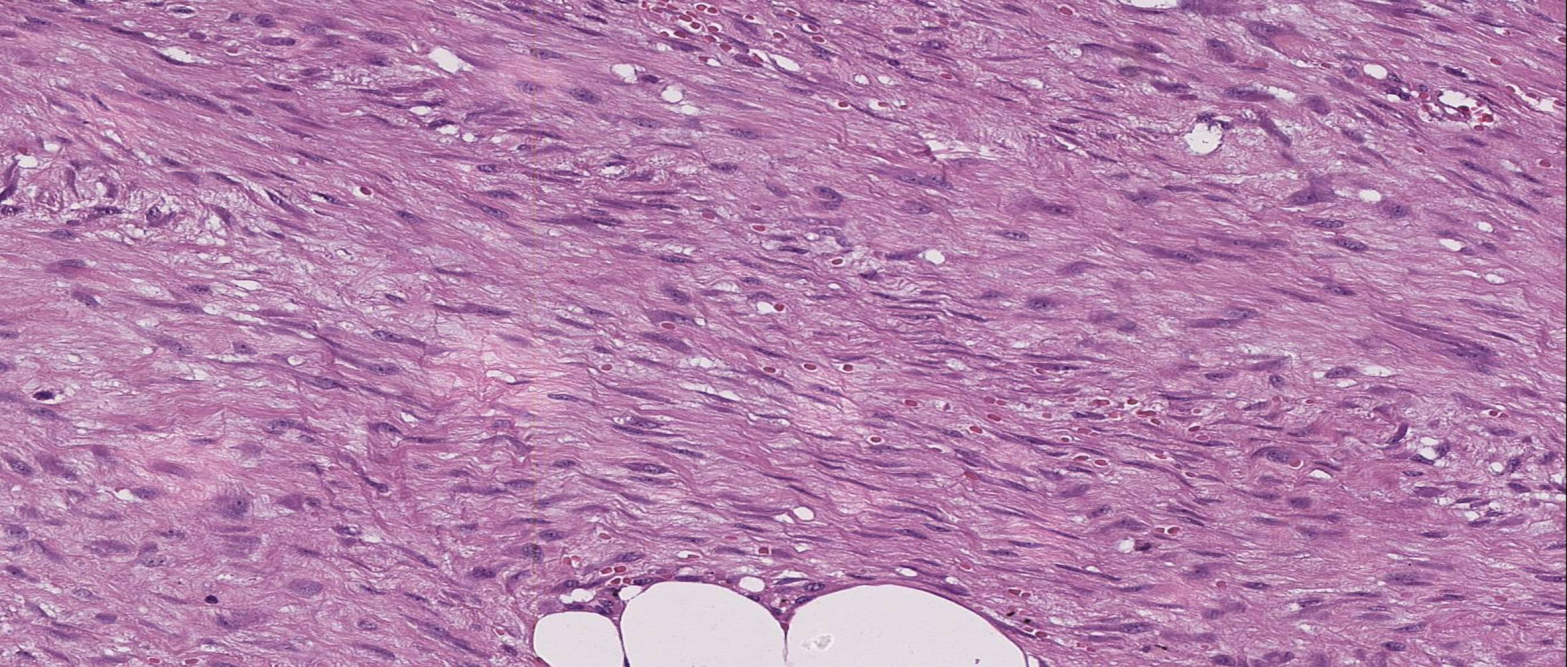
The neoplastic lesion is composed of irregular and long fascicles consisting of fibroblasts and myofibroblasts accompanied with few capillaries with thin walls (H&E, 100x).

**Figure 3:**
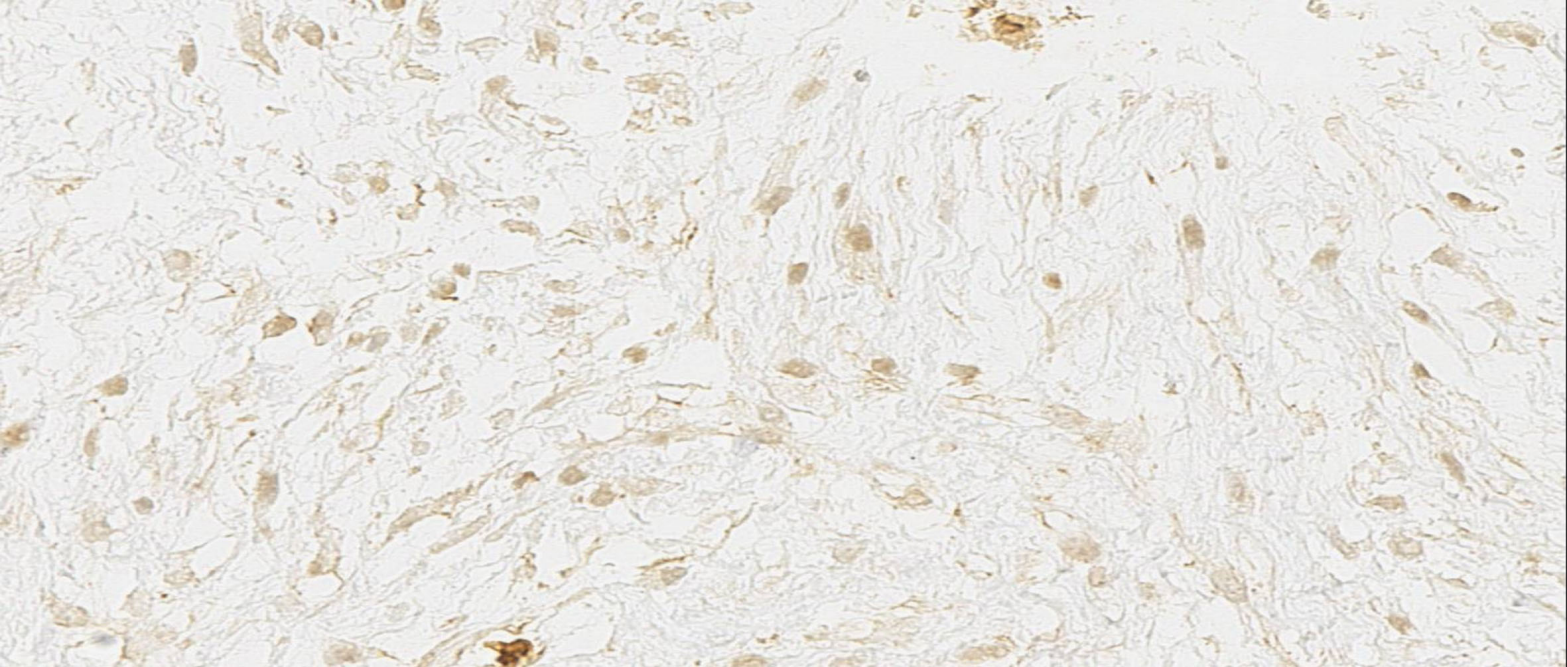
Immunohistochemistry: The nucleus shows β-catenin expression in the neoplastic cells (400x).

**Figure 4:**
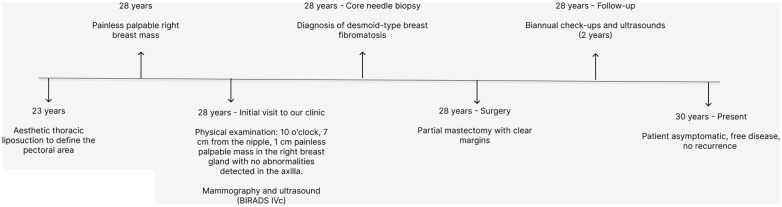
Timeline: patient evolution.

Mammography and ultrasound reported BIRADS IVc corresponding to a solid, oval-shaped, isodense nodule with a dark halo and indistinct margins measuring 2.3 cm. The imaging findings closely resembled a malignant breast tumor, highlighting the diagnostic challenges posed by desmoid-type fibromatosis, which can mimic carcinoma in imaging studies. A biopsy was taken by Tru-cut of the lesion, which reported a mesenchymal lesion composed of spindle cells with oval nuclei with fine chromatin and small nucleoli. No evidence of atypia, necrosis, or mitosis was found. No evidence of mammary tissue was present. Compatible with desmoid-type breast fibromatosis (**Figures 1**, **2**), positive beta-catenin by immunohistochemistry (**Figure 3**). A partial mastectomy was performed to remove the lesion, with clear margins reported. He is currently disease free and has been followed up biannually for the past two years, with no evidence of recurrence. This case emphasizes the importance of early recognition and surgical intervention in managing desmoid-type fibromatosis, particularly in rare presentations such as this in a male patient following cosmetic surgery. Routine imaging studies, including ultrasound, were performed at each follow-up visit to monitor the surgical site, and all results showed no new masses or abnormalities. Physical examinations were also conducted at each visit, confirming the absence of any clinical signs of recurrence. The patient remains asymptomatic and in good health to date (**Figure 4**).

## 3 Discussion

This 28-year-old male patient underwent a liposuction procedure for aesthetic reasons 5 years prior to developing a palpable, firm mass. The mass was later diagnosed as desmoid-type breast fibromatosis. It originates from fibroblastic and myofibroblastic cells and is commonly found originating from the pectoralis major fascia and expanding to the breast (2). Surgery is a common factor associated with breast fibromatosis (2). Trauma, silicone implants, Gardner’s Syndrome, and Familial Adenomatous Polyposis are other entities related to this tumor (1).

The average size of the breast fibromatosis tumors is 2.5 to 3.0 cm, with reports of 10 cm at its highest (3). It presents with clinical characteristics similar to breast carcinoma, such as a firm palpable mass with dimpling, skin retraction (2). A key difference being that fibromatosis doesn’t present with nipple discharge or adenopathy (4). The diagnostic approach warrants imaging studies. On ultrasound breast fibromatosis is seen as an irregular hypoechoic mass with parallel orientation; it does not have acoustic shadowing, echogenic halo, or microcalcification, unlike breast cancer (2, 3). A contrast-enhanced MRI is helpful to differentiate breast fibromatosis from breast carcinoma. It also helps to evaluate the tumor extent for preoperative planning (4).

Owing to its clinical and radiological characteristics, a firm mass with indistinct margins requires a histological report to rule out first the presence of malignancy. The main neoplastic differential diagnosis include breast cancer and several types of breast sarcomas (5). Similarly, primary breast lymphoma, although rare, can occur in men and mimic carcinoma in imaging studies, highlighting the need for an accurate diagnosis to avoid unnecessary treatments (6). Phyllodes tumor, a lesion that akin to desmoid tumors also originates from connective tissue, represents a generally benign neoplasm with potential for malignant transformation that shares some similarities to a desmoid tumor’s presentation (7). These tumors have an association with breast trauma, are often voluminous, exhibit rapid growth and can mimic malignancy (7). Other benign differential diagnoses include nodular fasciitis, hypertrophic scarring, myofibroblastic pseudotumor, leiomyoma, lipoma, and benign fibroblastic spindle cell tumors (5, 8).

A breast biopsy is the diagnostic approach for breast fibromatosis confirmation. There are no specific immunomarkers for breast fibromatosis although our patient presented with β-catenin which is useful for establishing the diagnosis as somatic β-catenin-activating mutations are considered to be the cause of the disease (9). Accumulation of β-catenin is present in 82% of breast fibromatosis (10). There were no signs of cellular atypia. This is an important sign to differentiate breast fibromatosis from fibromatosis-like spindle cell metaplastic carcinoma. It is reported that cellular atypia is a sign that aligns with fibromatosis-like spindle cell metaplastic carcinoma, especially those with dark hyperchromatic pleomorphic nuclei, which are normally not present in breast fibromatosis (11).

The available treatment options were thoroughly reviewed with the patient, who expressed a preference to undertake a definitive approach over conservative management, opting for local excision of the tumor mass. The patient expressed a preference for this therapeutic option for it’s relatively low recurrence rate and immediate effect (2). Wide local excision with satisfactory margins is the main treatment of breast fibromatosis, as the tumor recurrence rate ranges from 18-29% (2). There are reports that defend a conservative approach, as surgical excision is associated with a high rate of re-excision (33–46%) and the rate of recurrence is not considerably lower than the surveillance group (12%) (6). On the other hand, surveillance leads to spontaneous regression in only 28–50% of cases of extra-abdominal fibromatosis (12). Patients who are not surgical candidates may receive other treatment options such as radiation and Tamoxifen in combination with anti-inflammatories (2, 4). NSAID action is related to the Wnt/β-catenin pathway, via cyclooxygenase-2 (COX-2), whose role in breast fibromatosis has been thoroughly mentioned (10). Tyrosine-kinase inhibitors is another option that has shown partial response, although the exact mechanism through which it acts has not been established (10).

This case is particularly notable due to the extremely rare presentation of desmoid-type breast fibromatosis in a male patient following an aesthetic liposuction procedure. While breast fibromatosis itself is uncommon, its occurrence in men is even rarer, with very few cases reported in the literature. This case represents the first reported occurrence of desmoid-type fibromatosis in the breast of a male patient in Mexico. In contrast to previously reported cases, our patient is significantly younger and has a notable history of cosmetic surgery, a risk factor that was not identified in other cases. For instance, in the case reported by Portela Melo et al. (2020) from Brazil, a 65-year-old male developed a breast mass without any familial or surgical history, and underwent a partial resection with clear margins (13). Similarly, Khalid et al. (2023) described a 49-year-old male from Pakistan, also without known risk factors, who developed a breast mass over 10 months and was treated with wide local excision (14). In Belgium, Roman et al. (2019) reported a case of a 66-year-old male with a genetic mutation (APC) who underwent mastectomy due to the tumor’s size and muscle infiltration (4). Unlike these cases, our patient developed the mass five years after a cosmetic surgery, suggesting a potential link between surgical trauma and the development of fibromatosis. The treatment involved a partial mastectomy with clear margins, and no recurrence has been observed after two years of follow-up. This case highlights the importance of early diagnosis, as desmoid fibromatosis, as seen in the reported cases, can mimic breast carcinoma in imaging.

The association with a prior cosmetic procedure further distinguishes this case, as it highlights the potential link between surgical trauma and the development of fibromatosis in the breast, an area where few such reports exist. This underscores the importance of considering benign fibromatosis in the differential diagnosis of breast masses, even in male patients and in the context of prior cosmetic surgeries.

## 4 Conclusion

In conclusion, this case of desmoid-type breast fibromatosis in a male patient following cosmetic liposuction highlights the rarity of this benign but locally aggressive condition, particularly in men. The case underscores the importance of considering fibromatosis in the differential diagnosis of breast masses, especially when imaging suggests malignancy, to avoid unnecessary interventions. This case adds to the limited number of reported instances and provides insight into the diagnostic and therapeutic approach to such rare presentations.

## Data Availability

The raw data supporting the conclusions of this article will be made available by the authors, without undue reservation.
